# Serum exosomal miR-141-3p and miR-3679-5p levels associated with endotype and postoperative recurrence in chronic rhinosinusitis with nasal polyps

**DOI:** 10.1016/j.waojou.2024.100938

**Published:** 2024-07-24

**Authors:** Gang Wang, Zizhen Liu, Jiabin Zhan, Rui Li, Yi Ye, Yanyan Qi, Xin Wei, Jing Zheng

**Affiliations:** Department of Otorhinolaryngology-Head and Neck Surgery, Hainan General Hospital, Hainan Affiliated Hospital of Hainan Medical University, Haikou, Hainan, China

**Keywords:** Exosomes, Nasal polyps, Biomarkers, Recurrence

## Abstract

**Background:**

Chronic rhinosinusitis with nasal polyps (CRSwNP) is a chronic inflammatory disease. Exosomes were involved in different inflammatory diseases, but their roles in CRSwNP were poorly explored.

**Method:**

We collected serum samples from 8 CRSwNP patients and 8 healthy controls (HC) and isolated their exosomes. MiRNA sequencing was performed for the exosome samples and differentially expressed miRNAs were identified. The top 3 differentially expressed exosomal miRNAs were confirmed in 2 validation cohorts, and their diagnostic values, predictive values for eosinophilic endotype, and recurrence were evaluated.

**Results:**

Distinctive serum exosomal miRNA profiles were observed between CRSwNP and HC groups. Reverse transcription-polymerase chain reaction results in the first validation cohort revealed that serum exosomal miR-141-3p levels were increased, and miR-18a-5p and miR-3679-5p levels were decreased in the CRSwNP group compared to the HC group. These 3 miRNAs were further validated in the second validation cohort, and the results showed that miR-141-3p levels were elevated and miR-3679-5p levels were reduced in the serum exosomes in the eosinophilic CRSwNP group in comparison with the non-eosinophilic CRSwNP group. Receiver operating characteristic (ROC) curves highlighted that exosomal miR-141-3p and miR-3679-5p exhibited promising values for predicting the eosinophilic endotype. The patients in the second cohort were followed up for 2 years, and categorized into recurrence and non-recurrence groups. The serum exosomal miR-141-3p levels were increased and miR-3679-5p levels were reduced in the recurrence group in comparison with the non-recurrence group. ROC curves and Kaplan-Meier survival analysis revealed significant associations between the levels of exosomal miR-141-3p and miR-3679-5p and the risk of postoperative recurrence.

**Conclusions:**

This study identified unique miRNA expression patterns in serum exosomes of CRSwNP patients. Circulating exosomal miR-141-3p and miR-3679-5p emerged as novel biomarkers for diagnosing CRSwNP, predicting the eosinophilic endotype, and forecasting postoperative recurrence.

## Introduction

Chronic rhinosinusitis with nasal polyps (CRSwNP) is a heterogeneous inflammatory disease of the airways, and it has affected more than 12% of the general population.^1^, ^2^, ^3^ The pathogenesis of CRSwNP remains unclear, and the accumulation of inflammatory cells and cytokines mediating changes in the tissue immune microenvironment plays a significant role in its pathological mechanisms.^2^^,^^4^^,^^5^ Although existing drug therapies and surgical interventions can improve clinical symptoms and quality of life in CRSwNP, the complex pathogenesis, and high tissue heterogeneity contribute to suboptimal treatment outcomes and a relatively high recurrence rate post-treatment.^6^^,^^7^ Recently, researchers have been conducting endotypic classification of CRSwNP to obtain targeted treatments.^8^, ^9^, ^10^ Currently, the most common endotype is the eosinophilic subtype, which is classified based on the degree of tissue eosinophil infiltration into eosinophilic CRSwNP (eCRSwNP) and non-eosinophilic CRSwNP (neCRSwNP).^11^, ^12^, ^13^ Therefore, screening for objective biomarkers to predict eCRSwNP can better achieve precision treatment, adjust follow-up protocols, and prevent postoperative recurrence.

Exosomes, small vesicles enveloped by a bilayer lipid membrane, are actively secreted by various cell types and are abundant in different bodily fluids.^14^^,^^15^ They play a crucial role in inter-cellular communication by transporting bioactive molecules involved in immune response modulation, tissue repair, and cellular homeostasis regulation.^16^ MicroRNAs (miRNAs) are particularly noteworthy among the diverse cargo carried by exosomes, being extensively expressed and studied molecules.^17^ Recent evidence indicates the significant potential of circulating exosomal miRNAs as non-invasive biomarkers for disease diagnosis, prognosis, and recurrence prediction.^18^^,^^19^ These characteristics have been confirmed in inflammatory airway diseases.^20^^,^^21^ Researchers have observed that the expression profiles of serum exosome-carried miRNAs are involved in the occurrence and development of asthma, the severity of allergic rhinitis, and the effectiveness of antigen-specific immunotherapy.^20^^,^^22^ However, there is currently no reported information on the expression profile and role of circulating exosomal miRNAs in CRSwNP, as well as their potential as indicators for CRSwNP diagnosis and predicting tissue eosinophilic endotype and recurrence.

In this study, our objective was to examine the signatures of serum exosomal miRNAs in CRSwNP patients and healthy controls (HC). We identified the differentially expressed exosomal miRNAs and validated them in 2 independent cohorts. We evaluated the diagnostic significance, the predictive value for eosinophilic endotypes, and the recurrence rates associated with these differentially expressed exosomal miRNAs. The findings from this study offer new insights into the diagnosis and targeted interventions for CRSwNP.

## Methods

### Patients and settings

In this study, we included 8 CRSwNP patients and 8 HCs in the discovery cohort. The first validation cohort includes 30 CRSwNP patients and 30 HCs, and the second validation cohort consists of 38 eCRSwNP patients and 38 neCRSwNP patients. All participants in this study were enrolled in our department between May 2021 and July 2021. CRSwNP was diagnosed according to the European Position Paper on Rhinosinusitis and Nasal Polyps 2012.^23^ Patients with incomplete clinical data, fungal sinusitis, allergic fungal rhinosinusitis, sinonasal tumors, recent oral corticosteroid or leukotriene modifier or antibiotic use within 4 weeks before recruitment, acute inflammation, asthma, or age below 18 or above 65 years were excluded from the study. Healthy volunteers without nasal inflammatory diseases or other inflammatory conditions were included as healthy controls (HCs). HCs were excluded if they were currently using or had received systemic steroids, antibiotics, or anti-allergy treatment within the past 4 weeks. This study was approved by the ethical committee in our hospital. All participants signed informed consent.

### Definition of eosinophilic CRSwNP

In the second validation cohort, nasal polyp samples were collected during functional endoscopic sinus surgery (FESS), then fixed, and sectioned for routine hematoxylin and eosin (HE) staining. The presence of tissue eosinophils (EOS) was determined by counting positively stained cells in representative high-power fields (HPFs) under a microscope. EOS count was quantified by averaging counts from 5 randomly selected HPFs, and the EOS percentage was defined as the ratio of the EOS count to the total number of inflammatory cells. Patients with tissue EOS percentages greater than 10% were classified as eosinophilic CRSwNP, while those with percentages below 10% were categorized as non-eosinophilic CRSwNP, as previously described.^8^^,^^24^^,^^25^

### Patient follow-up and definition of recurrence

All CRSwNP patients in the second validation cohort received FESS performed by 3 experienced surgeons and followed up for at least 2 years. All patients received standardized postoperative management, including postoperative nasal cleaning, nasal endoscopic examination, nasal saline irrigation, and nasal spray with steroids. For patients with poorly controlled postoperative symptoms, oral broad-spectrum antibiotics were administered for 1–2 weeks. After a two-year follow-up, patients were classified into recurrent and non-recurrent groups based on persistently disabling symptoms, endoscopic indicators, and/or computed tomography (CT) findings for a minimum of 2 months, despite the administration of the antibiotic and oral steroid rescue regimen described previously.^6^^,^^26^

### Demographic data and sample collection

Detailed demographic and clinical variables, including gender, age, body mass index (BMI), accompanying disease, Lund-MacKay and Lund-Kennedy scores, tissue, and peripheral blood EOS count, and percentages were collected from all subjects (including HCs and CRSwNP patients) before their recruitment in all cohorts. Ten milliliters of fasting blood were collected from each participant using a vacuum blood collection tube. The collected blood was then centrifuged at 2000 g for 10 min to isolate serum samples. The obtained serum samples were promptly stored at −80 °C for subsequent experiments.

### Serum exosome isolation and identification

The serum exosomes were extracted using the Total Exosome Isolation Reagent kit (Invitrogen®, USA) as previously described.^22^ Subsequently, the extracted exosomes were used for either identification or RNA extraction. After purifying the exosomes, we characterized their morphology using transmission electron microscopy (TEM) following established protocols. The particle size and size distribution of the collected exosomes were determined using dynamic light scattering. Western blotting was conducted to examine the protein levels of specific exosome markers in both exosome samples and serum without exosome samples in the discovery cohort. Specific primary antibodies for exosome markers, including CD63 (Abcam, UK) and CD81 (Abcam, UK), along with secondary antibodies (Abcam, UK), were employed as previously described.^22^^,^^27^

### RNA extraction and miRNA-sequencing

The total RNA containing miRNAs was extracted from exosomes collected from CRSwNP patients and HCs in the discovery cohort, and the concentration and purity were evaluated. Following RNA extraction, a sequencing small RNA library was constructed utilizing TruSeq Small RNA Sample Prep Kits (Illumina, USA) according to established protocols as previously described.^27^^,^^28^ This procedure included reverse transcription, cDNA synthesis, adapter addition, and sequencing primer ligation. Subsequently, miRNA sequence analysis was conducted using the Illumina NextSeq 500 platform (Illumina, USA).

### Data analysis and target prediction

The initial sequencing data underwent stringent quality control, and unqualified reads were filtered out to ensure the acquisition of clean data. The cleaned data were aligned against the miRNA sequence from an online database and subsequently analyzed using the statistical R package. Differentially expressed miRNAs were identified based on a fold change (FC) ≥1.5 or FC ≤ 0.67 and a *P*-value <0.05, comparing the CRSwNP and HC groups. Visualization tools such as heatmap analysis and volcano plots were employed to depict the differentially expressed miRNAs. Additionally, the Kyoto Encyclopedia of Genes and Genomes (KEGG) pathway enrichment analysis was performed to predict the function of the enriched target genes associated with the differentially expressed miRNAs.

### Exosomal miRNA quantification using RT-PCR

To confirm the sequencing results, we chose the 3 most significantly dysregulated exosomal miRNAs for verification through reverse transcription-polymerase chain reaction (RT-PCR) in 2 independent cohorts. In this process, total RNA was extracted from exosomes, and cDNA synthesis was performed using total RNA and the Mir-X miRNA First-Strand Synthesis Kit (Takara, China) referring to the provided instructions. Subsequently, quantitative PCR was employed to amplify and quantify the cDNA molecules corresponding to the target miRNAs, utilizing the miRNA primers outlined in Table S1. The relative expression levels of miRNAs were normalized to the external control cel-miR-39 using the 2^−ΔΔCT^ method, as previously described.^11^^,^^22^

### Statistical analysis

Normally distributed continuous variables will be presented as mean ± standard error and analyzed using Student's t-test. For variables lacking a normal distribution, median and interquartile ranges (IQRs) will be provided, and the Mann-Whitney *U* test will be applied for comparisons. Categorical data will be presented as frequencies and percentages, and comparisons will be conducted with a chi-square test. The diagnostic and predictive values of exosomal miRNAs will be assessed using receiver operating characteristic (ROC) curves. Correlation analysis will be conducted to explore the relationship between serum exosomal miRNA levels and tissue as well as peripheral blood eosinophilic inflammation. CRSwNP patients will be stratified into high and low serum exosomal miRNA groups according to the median values of serum exosomal levels. Kaplan-Meier survival analysis will be performed to examine their associations with the risk of postoperative recurrence. Data analysis will be carried out using SPSS 22.0, and GraphPad Prism 7.0 will be utilized for data analysis and graphing. Statistical significance will be defined as a two-tailed *P*-value <0.05.

## Results

### Identification of isolated serum exosomes

**Fig. S1:**
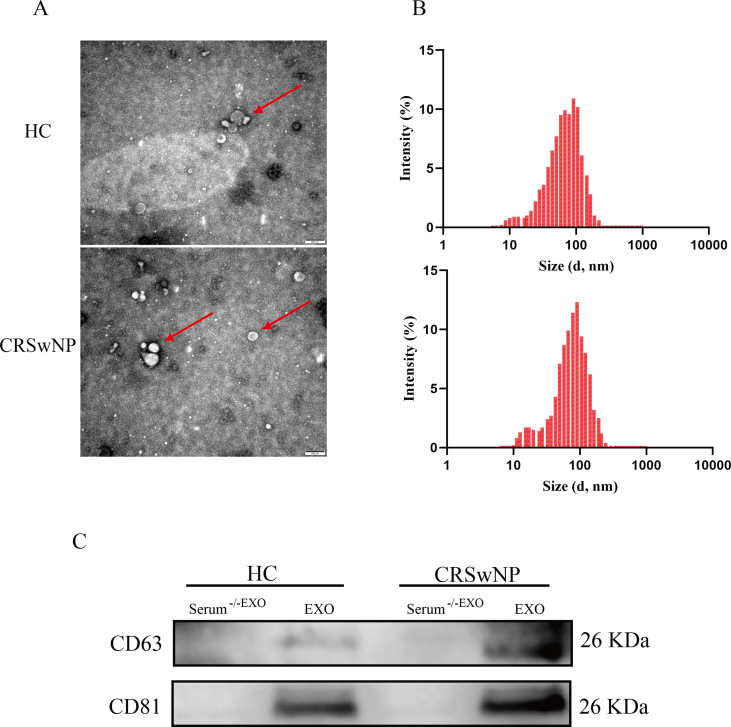
Characterization and identification of serum EXO in HCs and CRSwNP patients in the discovery cohort. (A) Electron microscopic images of serum EXO. Scale bar = 100 nm. (B) Nanoparticle tracking analysis of the size distribution of serum EXO. (C) Immunoblotting analysis of CD63 and CD81 in EXO and serum without EXO. EXO, exosome; HC, healthy control; CRSwNP, chronic rhinosinusitis with nasal polyps.

Serum exosomes were characterized based on their morphology, diameter, and surface proteins in the discovery cohort. TEM images exhibited the typical round cuplike morphology of the isolated exosomes from both CRSwNP and HC groups (Fig. S1A). Results from nanoparticle tracking analysis in Fig. S1B indicated that the particle diameters were approximately 100 nm. WB images demonstrated positive expressions of CD81 and CD63 in the detected exosome samples, while negative expression was observed in serum samples without exosomes (Fig. S1C). These findings collectively indicate the successful collection of exosomes from both groups for subsequent sequencing and validation.

### Serum exosomal miRNA profiles between CRSwNP patients and HCs

**Fig. 1:**
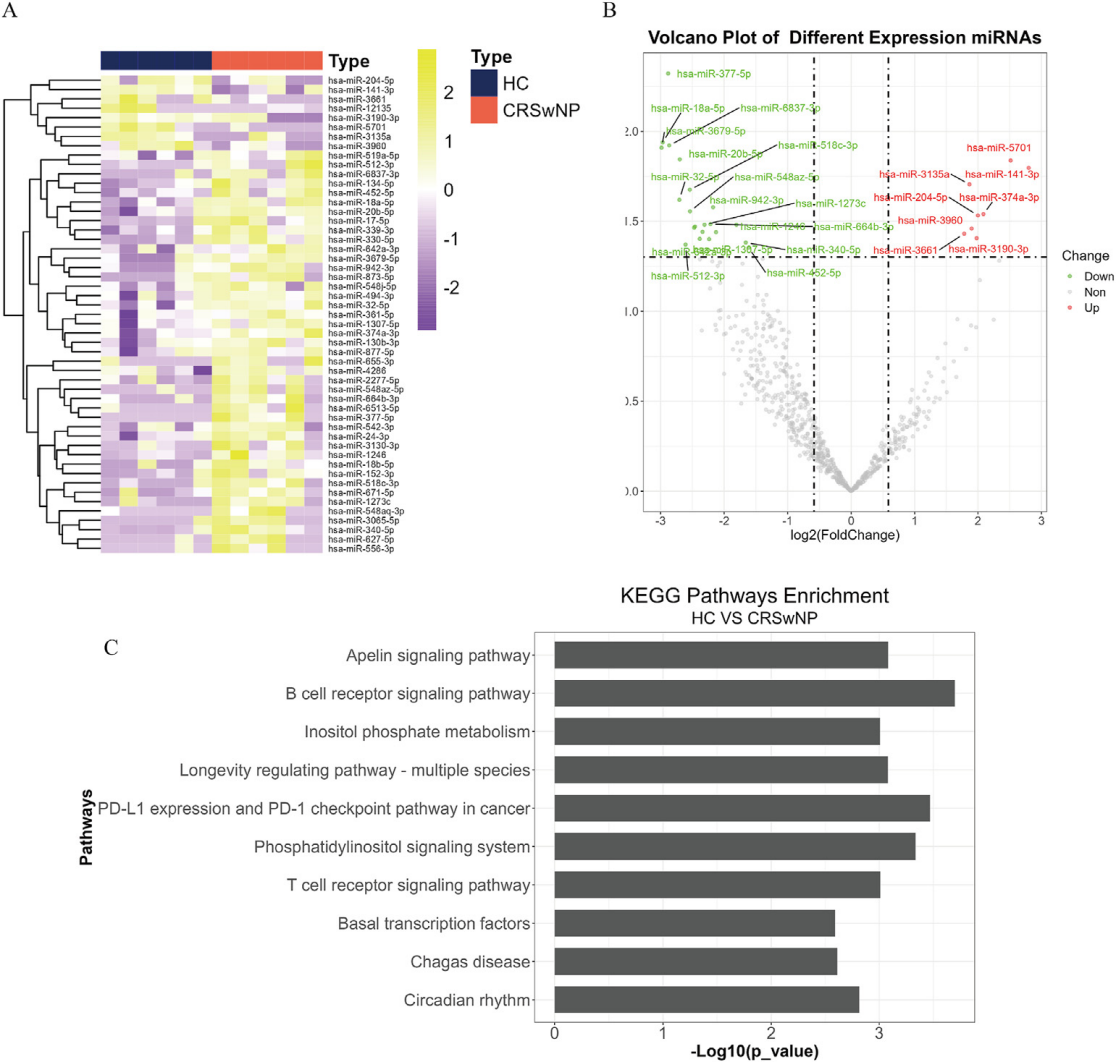
Landscape of exosomal miRNA profiles between HC and CRSwNP groups in the discovery cohort. (A) The heat map shows the exosomal miRNA profiles between HC and CRSwNP groups. (B) The volcano plot of differentially expressed serum exosomal miRNAs. (C) The top 10 most significant KEGG pathway terms of target genes. HC, healthy control; CRSwNP, chronic rhinosinusitis with nasal polyps. KEGG, kyoto encyclopedia of genes and genomes

In the discovery cohort, we recruited 8 CRSwNP patients and 8 HCs. The demographic characteristics of these participants are listed in Table S2. Serum exosome samples were collected from these subjects, and miRNA sequencing was performed. A total of 1071 miRNAs were identified in the serum exosomes between the 2 groups. The heat maps and volcano plots in Fig. 1A–B showed distinctive serum exosomal miRNA profiles between the 2 groups. Among them, 29 differentially expressed miRNAs were screened out, including 8 up-regulated and 21 down-regulated miRNAs, between the 2 groups. To elucidate the pathogenic significance of exosomal miRNAs in CRSwNP, we predicted potential target genes for the differentially expressed miRNAs. The KEGG pathway analysis Fig. 1C indicated that these miRNAs were prominently involved in pathways related to the B cell receptor and T cell receptor signaling pathways.

### Validation of differentially expressed serum exosomal miRNAs

**Fig. 2:**
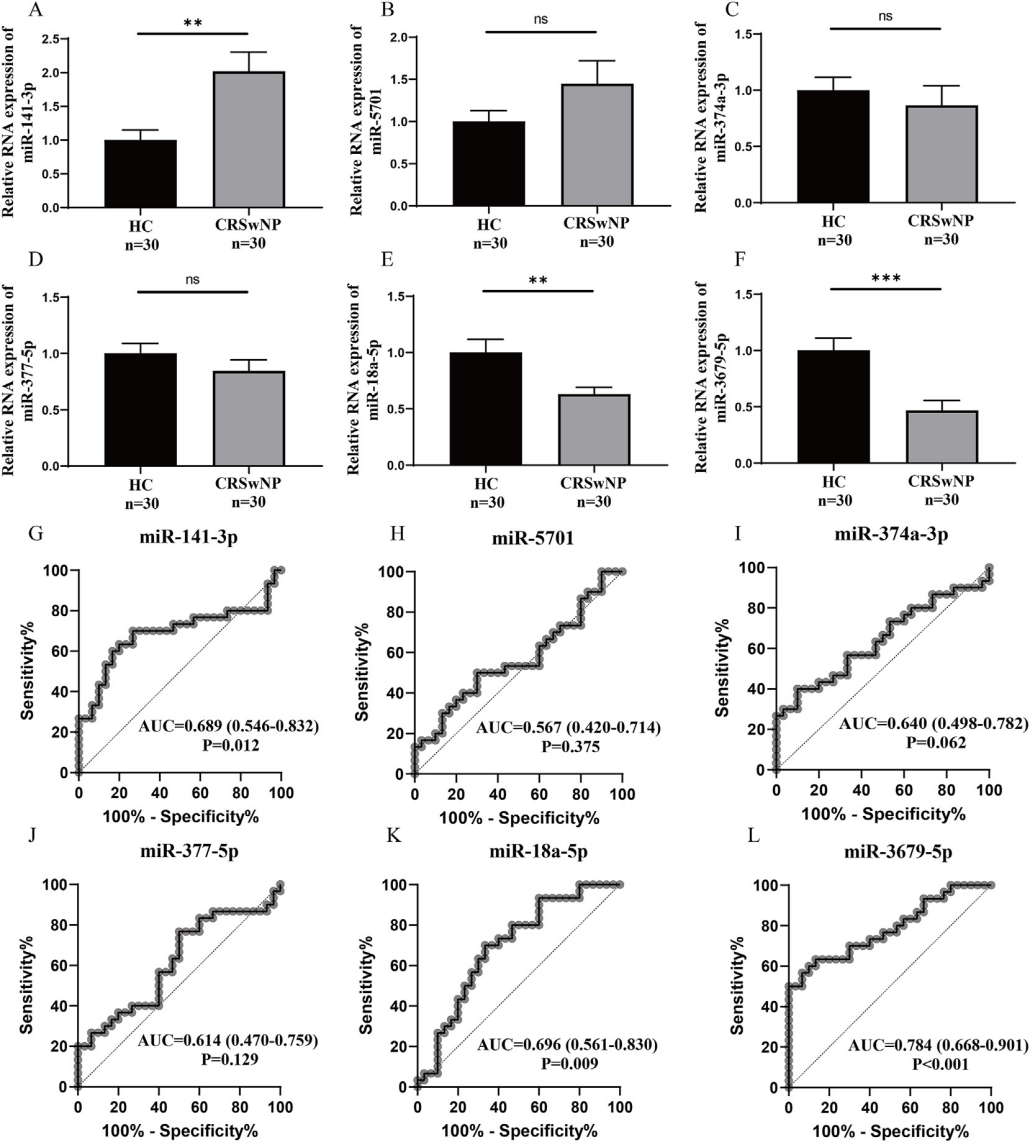
Validation of the top 3 most differentially expressed serum exosomal miRNAs in the first validation cohort. (A–F) comparison of the expressions of serum exosomal miRNAs between the HC and CRSwNP groups. (G–L) ROC curves show the diagnostic values of serum exosomal miRNAs for CRSwNP. HC, healthy control; CRSwNP, chronic rhinosinusitis with nasal polyps. ROC, receiver operating characteristic. ns, no significance; ∗∗P < 0.01; ∗∗∗P < 0.001

The top 3 significantly dysregulated serum exosomal miRNAs were chosen for validation in the first validation cohort. The detailed parameters of the top 3 significantly dysregulated miRNAs are listed in Table S3. The baseline data between the 2 groups in the first validation cohort are shown in Table 1. The RT-PCR results indicated elevated levels of miR-141-3p and reduced levels of miR-18a-5p and miR-3679-5p in serum exosomes from the CRSwNP group compared to those in the HC group. However, no statistical difference was found in serum exosomal miR-5701, miR-374a-3p, and miR-18a-5p between the 2 groups (Fig. 2A–F). ROC curves demonstrated that serum exosomal miR-141-3p, miR-18a-5p, and miR-3679-5p exhibited diagnostic values for CRSwNP (Fig. 2G–L).

**Table 1:** Demographic characteristics between HCs and CRSwNP patients in the first validation cohort.

	HC	CRSwNP	P
Number, n	30	30	
Male/female	21/9	17/13	0.422
Age, years	41.5 (28.8, 53.8)	40.0 (31.8, 49.0)	0.754
BMI, kg/m^2^	24.0 (20.8, 26.4)	24.8 (20.7, 26.5)	0.854
Allergic rhinitis, yes/no	0/30	8/22	0.005
Asthma, yes/no	0/30	5/25	0.052
Lund-MacKay score	–	12.0 (11.0, 14.0)	–
Lund-Kennedy score	–	7.0 (5.0, 8.0)	–
Tissue EOS count, n/HPF	–	8.0 (4.0, 23.0)	–
Tissue EOS percentage, %	–	8.7 (5.5, 20.1)	–
Peripheral blood EOS count, 10^9^/L	0.2 (0.1, 0.3)	0.3 (0.2, 0.4)	0.002
Peripheral blood EOS percentage, %	3.5 (2.3, 4.8)	5.7 (3.8, 8.2)	<0.001

HC, healthy control; CRSwNP, chronic rhinosinusitis with nasal polyps; BMI, body mass index; EOS, eosinophil; HPF, high power field.

### Serum differentially expressed exosomal miRNAs associated with eosinophilic endotype

**Fig. 3:**
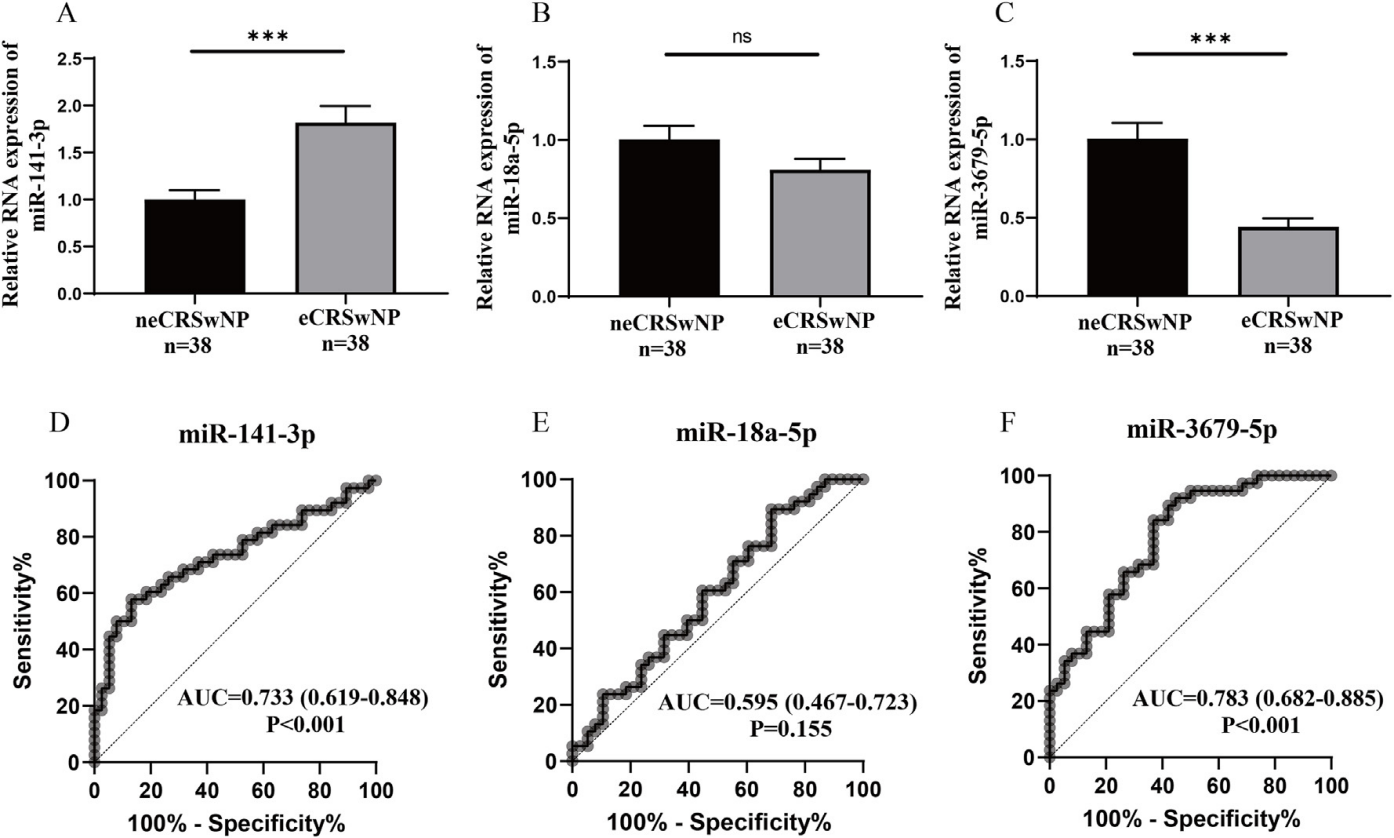
Exploration of expression levels of 3 candidate miRNAs in the serum EXO between the neCRSwNP and eCRSwNP groups in the second validation cohort. (A–C) Comparisons of serum exosomal miR-141-3p, miR-18a-5p, and miR-3679-5p expressions between the neCRSwNP and eCRSwNP groups. (D-F) ROC curves show the predictive values of serum exosomal miR-141-3p, miR-18a-5p, and miR-3679-5p for eCRSwNP. EXO, exosome; neCRSwNP, non-eosinophilic chronic rhinosinusitis with nasal polyps; eCRSwNP, eosinophilic chronic rhinosinusitis with nasal polyps. ns, no significance; ∗∗∗P < 0.001

To investigate the links between candidate serum exosomal miRNAs and the eosinophilic endotype in CRSwNP, we verified the expression of exosomal miR-141-3p, miR-18a-5p, and miR-3679-5p in the second validation cohort, which was independent from the first validation cohort, comprising 38 neCRSwNP patients and 38 eCRSwNP patients. The demographic and clinical data between the 2 groups in the second validation cohort are shown in Table 2. The rate of allergic rhinitis, Lund-MacKay score, tissue, and peripheral blood EOS count and percentage were higher in the eCRSwNP group compared to the neCRSwNP group. The results in Fig. 3A–C showed that serum exosomal miR-141-3p levels were increased, and miR-3679-5p levels were reduced in the eCRSwNP group in comparison with the neCRSwNP group. RCO curves highlighted that serum exosomal miR-141-3p and miR-3679-5p levels exhibited promising values for predicting eCRSwNP (Fig. 3D–F). Furthermore, the results of correlation analysis indicated a positive correlation between serum exosomal miR-141-3p levels and tissue EOS count and percentage, as well as peripheral blood EOS percentage. Conversely, miR-3679-5p levels showed a negative correlation with tissue and peripheral blood EOS count and percentage (Table S4).

**Table 2:** Demographic characteristics between neCRSwNP and eCRSwNP groups in the second validation cohort.

	neCRSwNP	eCRSwNP	P
Number, n	38	38	
Male/female	21/17	25/13	0.482
Age, years	42.0 (32.8, 51.3)	43.0 (30.0, 48.3)	0.644
BMI, kg/m^2^	22.1 (20.0, 25.4)	22.8 (20.5, 25.6)	0.827
Allergic rhinitis, yes/no	5/33	14/24	0.032
Asthma, yes/no	3/35	8/30	0.191
Lund-MacKay score	13.0 (10.8, 14.0)	14.0 (12.0, 16.0)	0.017
Lund-Kennedy score	6.0 (5.0, 8.0)	7.0 (6.0, 8.0)	0.114
Tissue EOS count, n/HPF	2.0 (1.0, 4.0)	10.5 (4.0, 31.0)	<0.001
Tissue EOS percentage, %	3.7 (1.2, 6.1)	23.7 (16.3, 36.0)	<0.001
Peripheral blood EOS count,10^9^/L	0.2 (0.1, 0.3)	0.3 (0.2, 0.5)	<0.001
Peripheral blood EOS percentage, %	3.2 (2.0, 5.3)	4.4 (2.9, 7.1)	<0.001

neCRSwNP, non-eosinophilic chronic rhinosinusitis with nasal polyps; eCRSwNP, eosinophilic chronic rhinosinusitis with nasal polyps; BMI, body mass index; EOS, eosinophil; HPF, high power field.

### Serum exosomal miR-141-3p and miR-3679-5p levels associated with postoperative recurrence

**Fig. 4:**
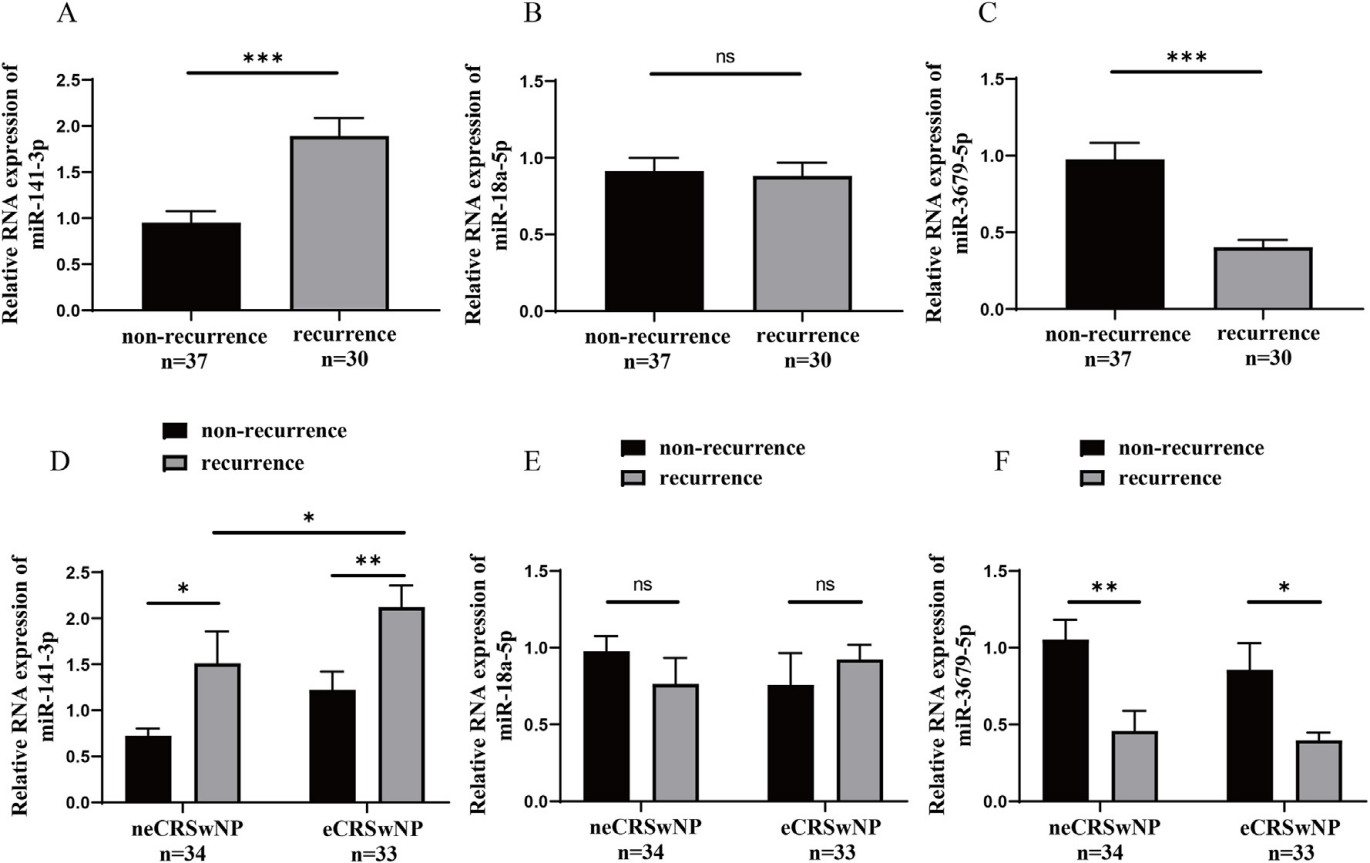
Exploration of expression levels of 3 candidate miRNAs in the serum EXO between the non-recurrence and recurrence groups. (A–C) Comparisons of serum exosomal miR-141-3p, miR-18a-5p, and miR-3679-5p expressions between the 2 groups. (D–F) The association between serum exosomal miR-141-3p, miR-18a-5p, and miR-3679-5p expressions and recurrence in the neCRSwNP and eCRSwNP groups. EXO, exosome; neCRSwNP, non-eosinophilic chronic rhinosinusitis with nasal polyps; eCRSwNP, eosinophilic chronic rhinosinusitis with nasal polyps. ns, no significance; ∗P < 0.05; ∗∗P < 0.001; ∗∗∗P < 0.001

**Fig. 5:**
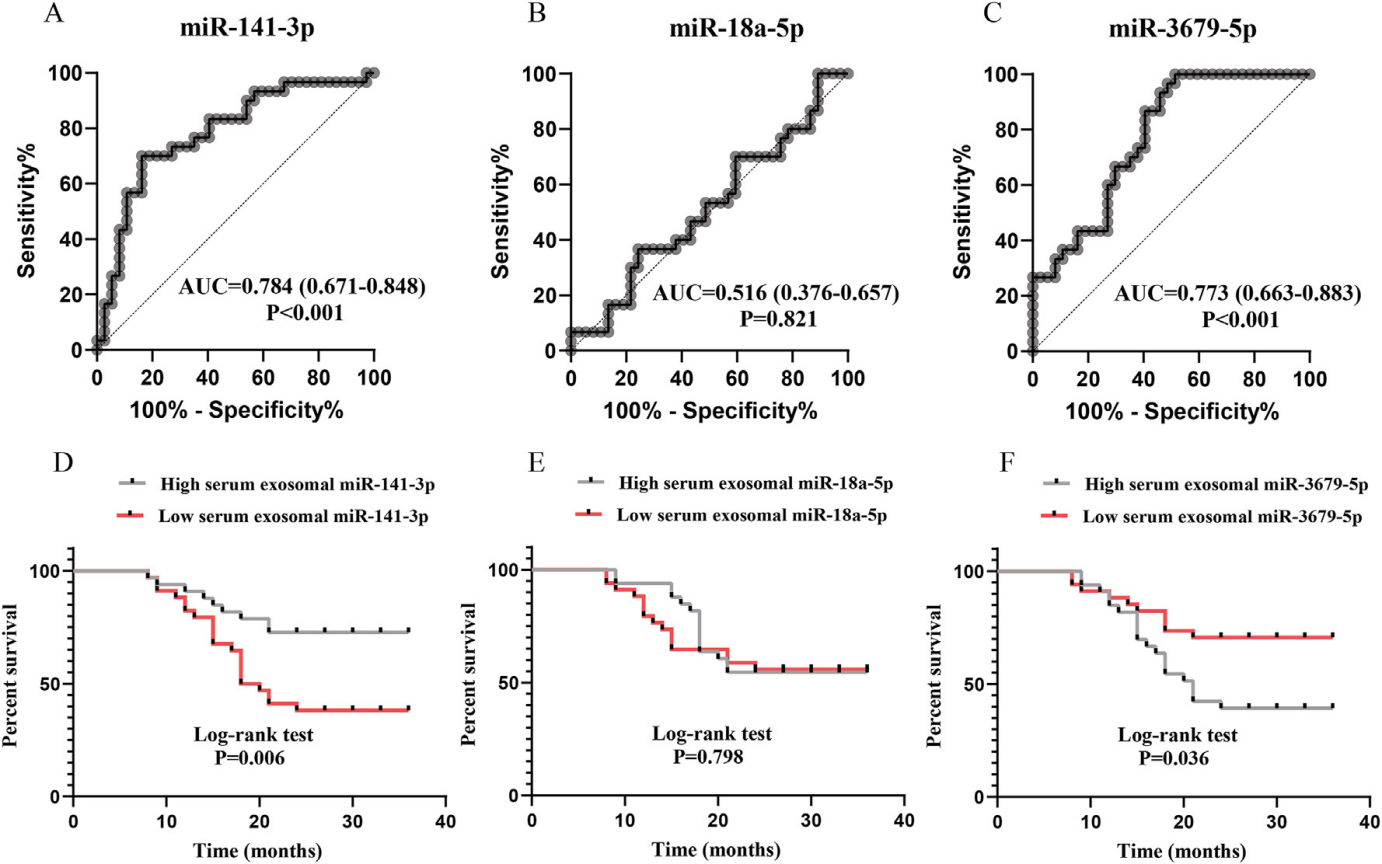
The predictive values of serum exosomal miR-141-3p, miR-18a-5p, and miR-3679-5p for postoperative recurrence. (A–C) ROC curves; (D–F) Kaplan-Meier survival analysis. ROC, receiver operating characteristic; AUC, the area under the curve

To validate the connections between these 3 serum exosomal miRNAs and the risk of postoperative recurrence, we conducted a follow-up of all CRSwNP patients in the second cohort for over 2 years to assess their prognosis. Finally, 67 patients completed the follow-up schedule, 37 patients were categorized into the non-recurrence group, and 30 patients were included in the recurrence group. Table 3 shows the demographic characteristics between the 2 groups. The tissue and peripheral blood EOS count and percentage, rate of eCRSwNP were higher, and the follow-up time was shorter in the recurrence group in comparison with the non-recurrence group. The results from RT-PCR showed an increase in serum exosomal miR-141-3p levels and a decrease in miR-3679-5p levels in the recurrence group compared to the non-recurrence group. These trends were consistently observed in both patients with eCRSwNP and neCRSwNP (Fig. 4). ROC and Kaplan-Meier survival analysis in Fig. 5 indicate an association between serum exosomal levels of miR-141-3p and miR-3679-5p and the risk of postoperative recurrence. Moreover, the unadjusted and adjusted Cox regression analysis suggested that serum exosomal miR-141-3p and miR-3679-5p levels were associated with the risk of postoperative recurrence in CRSwNP (Table S5). These findings suggest that the dysregulation of serum exosomal miR-141-3p and miR-3679-5p may be implicated in the mechanisms leading to recurrence in CRSwNP.

**Table 3:** Demographic characteristics between non-recurrence and recurrence groups in the second validation cohort.

	non-recurrence	recurrence	P
Number, n	37	30	
Male/female	21/16	19/11	0.625
Age, years	41.0 (32.5, 51.5)	43.0 (31.5, 49.3)	0.940
BMI, kg/m^2^	22.9 (20.6, 25.1)	22.5 (19.9, 25.7)	0.966
Allergic rhinitis, yes/no	6/31	12/18	0.051
Asthma, yes/no	4/33	6/24	0.324
Lund-MacKay score	13.0 (11.0, 15.0)	14.0 (10.0, 15.3)	0.787
Lund-Kennedy score	6.0 (5.0, 8.0)	7.0 (6.0, 8.0)	0.217
Tissue EOS count, n/HPF	3.0 (1.0, 5.5)	6.5 (2.0, 29.3)	<0.001
Tissue EOS percentage, %	5.6 (1.9, 16.3)	17.9 (4.2, 36.0)	<0.001
Peripheral blood EOS count,10^9^/L	0.2 (0.1, 0.3)	0.4 (0.3, 0.5)	<0.001
Peripheral blood EOS percentage, %	3.2 (1.9, 5.4)	6.3 (3.7, 8.5)	<0.001
neCRSwNP/eCRSwNP	26/11	8/22	0.001
Follow-up time, months	27.0 (24.0, 31.5)	15.0 (12.0, 18.0)	<0.001

BMI, body mass index; EOS, eosinophil; HPF, high power field; neCRSwNP, non-eosinophilic chronic rhinosinusitis with nasal polyps; eCRSwNP, eosinophilic chronic rhinosinusitis with nasal polyps.

## Discussion

CRSwNP is a complex medical condition characterized by intricate pathological mechanisms, diverse tissue types, and an increased risk of postoperative recurrence, posing a significant clinical challenge.^26^^,^^29^ In recent years, substantial research has been devoted to unraveling the complexities of CRSwNP, with a focus on elucidating its origins, comprehending the underlying pathogenic mechanisms, discovering dependable objective biomarkers for diagnosis, distinguishing endotypes, and predicting the probability of postoperative recurrence.^30^^,^^31^ Although numerous recent studies have confirmed the potential of several biomarkers to distinguish tissue endotypes, particularly eosinophilic endotypes, and predict postoperative recurrence, the sensitivity and specificity of these markers still need improvement.^25^^,^^32^ Furthermore, the underlying mechanisms associated with these biomarkers have not been fully elucidated.

Exosomes are emerging as novel biomarkers, exerting a pivotal influence in the pathogenesis of various diseases by transporting various bioactive substances.^33^^,^^34^ Recently, accumulating evidence suggests a close association between the expression profiles of serum-derived exosomal miRNA and the risk and prognosis of various inflammatory diseases and allergic conditions.^34^, ^35^, ^36^ Zhang et al^22^ found that circulating exosomal miR-146a-3p levels were elevated in allergic rhinitis patients and correlated with disease severity. Another study highlighted that serum exosome-derived miRNA profiles were associated with the efficacy of subcutaneous immunotherapy in pediatric allergic rhinitis, and several miRNAs were involved in the process of immune tolerance induction.^11^ However, there is currently no relevant research exploring the miRNA profiles in serum-derived exosomes of CRSwNP patients and their potential values in predicting tissue endotype and postoperative recurrence.

In this study, we identified a distinct miRNA signature in serum exosomes that differentiates between CRSwNP patients and HCs. KEGG pathway analysis highlighted the prominent involvement of several miRNAs in the B cell receptor and T cell receptor signaling pathways. We validated the top 3 differentially expressed miRNAs and found that serum exosomal miR-141-3p, miR-18a-5p, and miR-3679-5p levels were elevated in the CRSwNP patients, and exhibited potential diagnostic values, suggesting that they have the potential to be used as a diagnostic biomarker for CRSwNP. Comprehensive evidence underscored substantial changes in the quantity and functionality of immune cells, particularly B cells and T cells, both in tissue and peripheral blood of individuals with CRSwNP.^37^, ^38^, ^39^ Additionally, the B cell receptor and T cell receptor signaling pathways played pivotal roles in the immunopathological mechanisms associated with CRSwNP.^40^, ^41^, ^42^ Previous research has confirmed that serum-derived exosomal miRNAs can be taken up by diverse immune cells, subsequently targeting specific genes, thus influencing the activity and migration of immune cells. This process is integral to immune regulation, inflammation modulation, tissue repair, and remodeling.^43^^,^^44^ Furthermore, immune cells, when stimulated by antigens and stimuli, can release exosomes carrying miRNAs. These exosomes eventually enter the peripheral blood and are detected as specific markers for certain pathological processes or diseases. Therefore, we have reason to believe that circulating exosomal miRNA expression profiles in CRSwNP patients are associated with its pathological mechanisms. It may potentially influence the development and prognosis of the disease by modulating the functionality of immune cells such as B cells and T cells.

To further investigate the associations between serum exosomal miRNAs and the endotype and prognosis of CRSwNP, we verified the expression levels of the previously mentioned 3 miRNAs in the second validation cohort. Our findings revealed alterations in serum exosomal miR-141-3p and miR-3679-5p levels in eCRSwNP and demonstrated correlations with both tissue and circulating EOS counts and percentages. Additionally, serum exosomal miR-141-3p and miR-3679-5p levels were identified as having the potential to predict the risk of postoperative recurrence. Currently, multiple studies have found that miR-141-3p can influence cell proliferation and modulate immune cell functions, playing a significant role in tumor immunity.^45^, ^46^, ^47^ Yang et al^48^ demonstrated that miR-141-3p could modulate the intracellular reactive oxygen species via targeting GLS1, then affect malignant phenotype and CD8^+^ T cell function in bladder cancer microenvironments.^48^ Recent research has revealed that miR-141-3p derived from exosomes can modulate the β-catenin and TGF-β signaling pathways, thereby promoting tissue remodeling and epithelial-mesenchymal transition.^49^^,^^50^ It has been reported that epithelial miR-141 can enhance goblet cell hyperplasia and the Th2 response, leading to increased production of eosinophil-promoting cytokines such as IL-5 and IL-13. This mechanism is involved in the pathogenesis of asthma.^51^ Accordingly, oxidative stress damage, tissue remodeling, and EMT can influence the repair of nasal mucosal epithelial cells and the maintenance of the normal mucosal barrier.^24^^,^^32^ Mucosal barrier damage and Th2-eosinophilic inflammation are important tissue pathological features in eCRSwNP and recurrent CRSwNP.^52^, ^53^, ^54^ Based on previous literature and research findings, we hypothesize that serum exosome-carried miR-141-3p may potentially influence the function of Th2 cells and the repair of nasal mucosal epithelial cells. Consequently, it could impact the infiltration of inflammatory cells and tissue remodeling within the nasal mucosa, mediating the tissue pathological changes and postoperative recurrence in CRSwNP.

Currently, there is scarce information available regarding the biological functions of miR-3679-5p, and only a few studies have confirmed its abnormal expression in both tumors and inflammatory diseases. In a recent study, it was shown that individuals with rheumatoid arthritis exhibited a decrease in serum levels of miR-3679-5p, and this reduction was correlated with both the severity of the disease and its prognosis.^55^ Higashijima et al.^56^ discovered that miR-3679-5p was a newly identified TNF-α-responsive microRNA, it could inhibit the coordinated demethylation of H3K9 and H3K27, potentially affecting Th1 inflammation and thereby mitigating inflammatory responses in endothelial cells. It is widely recognized that TNF-α facilitating Th1 and non-eosinophilic inflammation plays a pivotal role in the pathological mechanisms of CRSwNP.^57^^,^^58^ It can stimulate immune and inflammatory responses, disrupt the mucosal barrier and Th1/Th2 inflammation balance, and contribute to tissue remodeling in the nasal mucosa, consequently amplifying tissue immunopathological damage and the likelihood of postoperative recurrence.^59^^,^^60^ Interestingly, we found that the decrease in miR-3679-5p showed a greater predictive value for recurrence in non-eosinophilic than in eosinophilic CRSwNP. These results may indicate that miR-3679-5p can influence the development of non-eosinophilic inflammation by regulating the TNF-α signaling pathway, thereby affecting the risk of postoperative recurrence.

Our study possesses several limitations. Firstly, the sample size is relatively small, and all participants are recruited solely from a single medical center. Secondly, we validated only the top 3 differentially expressed miRNAs, and other miRNAs with differential expressions were not validated. However, this does not suggest that they lack research value in the diagnosis of CRSwNP and prediction of endotype and postoperative recurrence. Thirdly, the specific effects and mechanisms of candidate serum exosomal miRNAs in eCRSwNP and its recurrent process are not fully elucidated.

## Conclusion

This study identified unique miRNA expression patterns in serum exosomes of CRSwNP patients. Circulating exosomal miR-141-3p and miR-3679-5p were potential biomarkers for diagnosing CRSwNP, predicting the eosinophilic endotype, and predicting postoperative recurrence.

## Abbreviations

CRSwNP, chronic rhinosinusitis with nasal polyps; HC, healthy controls; ROC, receiver-operated characteristic; eCRSwNP, eosinophilic chronic rhinosinusitis with nasal polyps; neCRSwNP, non-eosinophilic chronic rhinosinusitis with nasal polyps; miRNA, microRNA; FESS, functional endoscopic sinus surgery; HE, hematoxylin and eosin; EOS, eosinophils; HPF, high-power field; CT, computed tomography; TEM, transmission electron microscopy; KEGG, Kyoto Encyclopedia of Genes and Genomes; RT-PCR, quantitative reverse transcription polymerase chain reaction; IQR, interquartile ranges; EXO, exosome.

## Funding source

This research was supported by the National Natural Science Foundation of China Regional Fund Program (82160210 and 82060184), the Natural Science Foundation of Hainan Province (822MS168 and 821MS131), Hainan Province Science and Technology Special Fund (ZDYF2022SHFZ049), Natural Science Research Project “Open Competition Mechanism” of Hainan Medical University (JBGS202109).

## Availability of data and material

All data generated or analyzed during this study are included in this published article and its Supplemental file. More related data of the current study are available from the corresponding author upon reasonable request.

## Author contribution

(I) Conception and design: Gang Wang, Xin Wei, and Jing Zheng.

(II) Administrative support: Gang Wang, Zizhen Liu, and Jiabin Zhan.

(III) Collection and assembly of data: Rui Li, Yi Ye, and Yanyan Qi.

(IV) Data analysis and interpretation: Gang Wang and Xin Wei,

(V) Manuscript writing: All authors.

(VI) Final approval of manuscript: All authors.

## Ethics approval

This study was approved by the ethical committee of Hainan General Hospital (Med-Eth-Re [2023] 417). All participants signed informed consent.

## Consent for publication

All authors have seen and approved the last version and agreed to the publication of the work.

## Declaration of competing interest

There are no patents, products in development, or marketed products to declare.
