# Supplementary material for: Serum exosomal miR-141-3p and miR-3679-5p levels associated with endotype and postoperative recurrence in chronic rhinosinusitis with nasal polyps
Source: World Allergy Organ J. 2024 Jul 24;17(8):100938. doi: 10.1016/j.waojou.2024.100938 (PMC11327455; doi:10.1016/j.waojou.2024.100938)
Supplement: Multimedia component 1 [file mmc1.docx]

| Name | Forward primer | Reverse primer |
| --- | --- | --- |
| miR-141-3p | GCGCGTAACACTGTCTGGTAA | AGTGCAGGGTCCGAGGTATT |
| miR-5701 | CGCGCGTTATTGTCACGTT | AGTGCAGGGTCCGAGGTATT |
| miR-374a-3p | CGCGCGCTTATCAGATTGTATT | AGTGCAGGGTCCGAGGTATT |
| miR-377-5p | GCGAGAGGTTGCCCTTGGT | AGTGCAGGGTCCGAGGTATT |
| miR-18a-5p | CGCGTAAGGTGCATCTAGTGC | AGTGCAGGGTCCGAGGTATT |
| miR-3679-5p | CGTGAGGATATGGCAGGGA | AGTGCAGGGTCCGAGGTATT |

Table S1. Primers of detected miRNAs
