# Supplementary material for: Serum exosomal miR-141-3p and miR-3679-5p levels associated with endotype and postoperative recurrence in chronic rhinosinusitis with nasal polyps
Source: World Allergy Organ J. 2024 Jul 24;17(8):100938. doi: 10.1016/j.waojou.2024.100938 (PMC11327455; doi:10.1016/j.waojou.2024.100938)
Supplement: Multimedia component 2 [file mmc2.docx]

|  | HC | CRSwNP | P |
| --- | --- | --- | --- |
| Number, n | 8 | 8 |  |
| Male/female | 5/3 | 4/4 | 1.000 |
| Age, years | 42.0 (33.0, 47.0) | 40.0 (30.0, 51.0) | 0.902 |
| BMI, kg/m^2^ | 23.5 (21.2, 26.2) | 24.3 (21.5, 26.0) | 0.799 |
| Allergic rhinitis, yes/no | 0/8 | 2/6 | 0.467 |
| Asthma, yes/no | 0/8 | 1/7 | 1.000 |
| Lund-MacKay score | - | 12.0 (11.0, 14.0) | - |
| Lund-Kennedy score | - | 7.0 (6.0, 8.0) | - |
| Tissue EOS count, n/HPF | - | 11.0 (7.0, 26.0) | - |
| Tissue EOS percentage, % | - | 7.6 (5.2, 27.9) | - |
| Peripheral blood EOS count, 10^9^/L | 0.2 (0.1, 0.3) | 0.2 (0.2, 0.5) | 0.215 |
| Peripheral blood EOS percentage, % | 2.7 (1.6, 4.0) | 4.3 (2.5, 6.6) | 0.094 |

Table S2 Demographic characteristics of HCs and CRSwNP patients in the discovery cohort

HC, healthy control; CRSwNP, chronic rhinosinusitis with nasal polyps; BMI, body mass index; EOS, eosinophil; HPF, high power field.
