# Supplementary material for: Serum exosomal miR-141-3p and miR-3679-5p levels associated with endotype and postoperative recurrence in chronic rhinosinusitis with nasal polyps
Source: World Allergy Organ J. 2024 Jul 24;17(8):100938. doi: 10.1016/j.waojou.2024.100938 (PMC11327455; doi:10.1016/j.waojou.2024.100938)
Supplement: Multimedia component 3 [file mmc3.docx]

| Name | FC | Log2FC | Change | P value |
| --- | --- | --- | --- | --- |
| miR-141-3p | 6.88 | 2.80 | Up-regulation | 0.016 |
| miR-5701 | 5.28 | 2.51 | Up-regulation | 0.015 |
| miR-374a-3p | 4.65 | 2.33 | Up-regulation | 0.021 |
| miR-377-5p | 0.18 | -2.88 | Down-regulation | 0.012 |
| miR-18a-5p | 0.17 | -2.97 | Down-regulation | 0.011 |
| miR-3679-5p | 0.16 | -2.99 | Down-regulation | 0.004 |

Table S3. The parameters of the top 3 up-regulated and down-regulated miRNAs

FC, fold change
