# Supplementary material for: Serum exosomal miR-141-3p and miR-3679-5p levels associated with endotype and postoperative recurrence in chronic rhinosinusitis with nasal polyps
Source: World Allergy Organ J. 2024 Jul 24;17(8):100938. doi: 10.1016/j.waojou.2024.100938 (PMC11327455; doi:10.1016/j.waojou.2024.100938)
Supplement: Multimedia component 4 [file mmc4.docx]

|  | miR-141-3p | |  | miR-18a-5p | | |  | miR-3679-5p | |
| --- | --- | --- | --- | --- | --- | --- | --- | --- | --- |
|  | r P | |  | r | | P | | r P | |
| Tissue EOS count, n/HPF | 0.363 | 0.031 |  | 0.026 | 0.812 | |  | -0.407 | 0.002 |
| Tissue EOS percentage, % | 0.391 | 0.012 |  | 0.045 | 0.778 | |  | -0.459 | 0.004 |
| Peripheral blood EOS count,10^9^/L | 0.189 | 0.265 |  | 0.115 | 0.616 | |  | -0.311 | 0.035 |
| Peripheral blood EOS percentage, % | 0.201 | 0.048 |  | 0.134 | 0.817 | |  | -0.298 | 0.019 |

Table S4 Correlations between serum exosomal miRNAs and tissue and circulating EOS in the first validation cohort

EOS, eosinophil; HPF, high power field.
