# Supplementary material for: Serum exosomal miR-141-3p and miR-3679-5p levels associated with endotype and postoperative recurrence in chronic rhinosinusitis with nasal polyps
Source: World Allergy Organ J. 2024 Jul 24;17(8):100938. doi: 10.1016/j.waojou.2024.100938 (PMC11327455; doi:10.1016/j.waojou.2024.100938)
Supplement: Multimedia component 5 [file mmc5.docx]

|  | miR-141-3p | |  | miR-18a-5p | |  | miR-3679-5p | |
| --- | --- | --- | --- | --- | --- | --- | --- | --- |
|  | HR (95%CI) | P |  | HR (95%CI) | P |  | HR (95%CI) | P |
| Unadjusted | 2.723  (1.252-6.098) | 0.009 |  | 0.912  (0.541-1.713) | 0.814 |  | 0.521  (0.318-0.808) | 0.002 |
| Model 1 | 2.128  (1.219-5.077) | 0.019 |  | 1.098  (0.767-2.014) | 0.633 |  | 0.603  (0.376-0.845) | 0.010 |
| Model 2 | 2.205  (1.301-6.121) | 0.022 |  | 0.898  (0.679-1.829) | 0.809 |  | 0.577  (0.401-0.823) | 0.017 |
| Model 3 | 1.915  (1.089-5.654) | 0.047 |  | 0.933  (0.589-1.989) | 0.775 |  | 0.489  (0.297-0.765) | 0.033 |

Table S5. Cox regression analysis of the effects of serum exosomal miRNAs on the risk of postoperative recurrence in the second validation cohort

HR, hazard ratio; CI, confidence interval; EOS, eosinophil.

Model 1: Adjusted for gender, age, BMI;

Model 2: Adjusted for gender, age, BMI, allergic rhinitis, asthma, Lund-MacKay score, Lund-Kennedy score, and follow-up time;

Model 3: Adjusted for gender, age, BMI, allergic rhinitis, asthma, Lund-MacKay score, Lund-Kennedy score, follow-up time, tissue EOS count and percentage, peripheral blood EOS count and percentage, and other two serum exosomal miRNAs.
